# Differences in protein expression in Alzheimer’s disease patients based on risk factors for amyloid‐related imaging abnormalities

**DOI:** 10.1002/alz.084579

**Published:** 2025-01-09

**Authors:** Marlies Oosthoek, Lisa Vermunt, Elena Raluca Blujdea, Sjors G.J.G. in 't Veld, Wiesje M. van der Flier, Frederik Barkhof, Marta del Campo, Martín Pucheu Avilés, Allerdien Visser, Martijn C. Schut, Everard G.B. Vijverberg, Charlotte Teunissen

**Affiliations:** ^1^ Neurochemistry Laboratory, Department of Laboratory Medicine, Vrije Universiteit Amsterdam, Amsterdam UMC, Amsterdam Netherlands; ^2^ Alzheimer Center Amsterdam, Department of Neurology, Vrije Universiteit Amsterdam, Amsterdam UMC, Amsterdam Netherlands; ^3^ Neurochemistry Laboratory, Department of Laboratory medicine, Vrije Universiteit Amsterdam, Amsterdam UMC location VUmc, Amsterdam Netherlands; ^4^ Translational AI in Laboratory Medicine, Department of Laboratory Medicine, Vrije Universiteit Amsterdam, Amsterdam UMC, Amsterdam Netherlands; ^5^ Alzheimer Center Amsterdam, Neurology, Vrije Universiteit Amsterdam, Amsterdam UMC location VUmc, Amsterdam Netherlands; ^6^ Queen Square Institute of Neurology and Centre for Medical Image Computing, UCL, London United Kingdom; ^7^ Department of Radiology and Nuclear Medicine, Vrije Universiteit Amsterdam, Amsterdam UMC, Amsterdam Netherlands; ^8^ Barcelonaβeta Brain Research Center (BBRC), Pasqual Maragall Foundation, Barcelona Spain; ^9^ Neurochemistry Laboratory, Department of Laboratory Medicine, Vrije Universiteit Amsterdam, Amsterdam UMC, Amsterdam, North Holland Netherlands

## Abstract

**Background:**

The exact mechanism underlying amyloid‐related imaging abnormalities (ARIA) is unknown. Several factors explain ARIA risk, including the presence of microbleeds, APOE4 carriership, and very low Aβ42 levels. The cerebrospinal fluid (CSF) proteome reflects ongoing mechanisms and, thereby, provides an accessible fluid to refine risk of ARIA development. In this explorative study, we aimed to determine how the CSF proteome relates to the ARIA risk profile and identify potential biomarkers for ARIA risk.

**Method:**

We included AD dementia patients who were Aβ42/tTau ratio positive (n=156; median age: 67 IQR:60‐72, 38% F, median MMSE‐score: 19.8 IQR:17‐24). We dichotomized all risk factors: microbleeds were divided in 0 (M^‐^, n=123) or >0 (M^+^, n=33), APOE4 status was divided in carriers (APOE4^+^, n=96) and non‐carriers (APOE4^‐^, n=60), CSF Aβ42 levels were separated based on the median (Low and High). We also defined a high and low risk profile: patients with M^+^APOE4^+^A^L^ (n=13) and M^‐^APOE4^‐^A^H^ (n=23). CSF proteomics were performed using antibody‐based proteomics (Olink), and we selected 288 proteins previously shown to be related to AD (del Campo et al., 2022). With linear regression analysis (with age and sex corrections), we compared protein levels per risk group.

**Result:**

We identified 20 proteins that were differentially expressed in M^+^ versus M^‐^, 7 proteins in APOE4^+^ versus APOE4^‐^, 3 proteins in Aβ42^L^ versus Aβ42^H^, and 49 proteins in M^+^APOE4^+^A^L^ to M^‐^APOE4^‐^A^H^. The top proteins include EGFR, CRTAC1, AXL, CNTN1, and TIMP4. Overall, almost all proteins were downregulated in the ARIA risk group and most proteins were not overlapping between risk groups.

**Conclusion:**

These results show that there are differences based on the ARIA risk profile. The differentiating proteins could provide extra information on the ongoing biological processes that increase ARIA risk and in the future aid to refine ARIA risk. Potentially, these results could aid in the development of a new biomarker for ARIA risk. Future analysis focus on further understanding the proteins involved and validating the findings.

